# Identification and characterization of epithelial cells derived from human ovarian follicular fluid

**DOI:** 10.1186/s13287-015-0004-6

**Published:** 2015-02-20

**Authors:** Dongmei Lai, Minhua Xu, Qiuwan Zhang, Yifei Chen, Ting Li, Qian Wang, Yimeng Gao, Chunsheng Wei

**Affiliations:** The Center of Research Laboratory, The International Peace Maternity and Child Health Hospital, School of Medicine, Shanghai Jiaotong University, Shanghai, 200030 China; State Key Laboratory of Cell Biology, Institute of Biochemistry and Cell Biology, Shanghai Institutes for Biological Sciences, Chinese Academy of Sciences, Shanghai, 200031 China; Eye and ENT Hospital, Fudan University, Shanghai, 200031 China

## Abstract

**Introduction:**

Follicular fluid is important for follicular development and oocyte maturation. Evidence suggests that follicular fluid is not only rich in proteins but cells. Besides oocytes, the follicular fluid contains granulosa, thecal, and ovarian surface epithelial cells, and both granulosa and thecal cells are well-characterized. However, epithelial cells in follicular fluid are poorly studied. This study aims to isolate and characterize *in vitro* epithelial cells that originate from human ovarian follicular fluid retrieved in the assisted fertilization program.

**Methods:**

Follicular fluid samples were collected from 20 women in the assisted reproduction program. Epithelial cells were characterized by flow cytometry assay, immunofluorescence staining, real-time PCR, and time lapse photography.

**Results:**

Epithelial cell cultures were established from 18 samples. A small population of epithelial cells expresses germ-line stem cell markers, such as octamer-binding transcription factor 4 (OCT4), NANOG, and DEAD box polypeptide 4 (DDX4). In the epithelial cell culture system, oocyte-like cells formed spontaneously *in vitro* and expressed the following transcription markers: deleted in azoospermia-like (DAZL), developmental pluripotency associated protein 3 stella-related protein (STELLA), zona pellucida gene family C (ZPC), Syntaptonemal complex protein (SCP), and growth and differentiation factor 9 (GDF9). Some of the oocyte-like cells developed a zona pellucida-like structure. Both the symmetric and asymmetric division split of epithelial cells and early developing oocytes were observed using time lapse photography. Cell colonies were formed during epithelial culturing, which maintained and proliferated in an undifferentiated way on the feeder layer and expressed some pluripotency markers. These colonies differentiated *in vitro* into various somatic cell types in all three germ layers, but did not form teratoma when injected into immunodeficient mice. Furthermore, these epithelial cells could be differentiated directly to functional hepatocyte-like cells, which do not exist in ovarian tissues.

**Conclusions:**

The epithelial cells derived from follicular fluid are a potential stem cell source with a pluripotent/multipotent character for safe application in oogenesis and regenerative medicine.

**Electronic supplementary material:**

The online version of this article (doi:10.1186/s13287-015-0004-6) contains supplementary material, which is available to authorized users.

## Introduction

The formation of mammalian ovarian follicular antrum and formation of follicular fluids are important processes in follicular development. The proportion of follicular fluid at maximum size varies from species to species. Generally, larger species such as human, bovine, ovine, equine, and porcine have larger follicles, with the fluid comprising a substantial proportion of the follicle volumes at ovulation (estimated at >95% in bovine). Smaller species such as rats and mice have smaller follicles with fractionally less follicular fluid [1].

As the follicle grows, follicular antrum expansion clearly requires remodeling. There are three ovarian functional somatic cell types involved in folliculogenesis remodeling: the ovarian surface epithelium (OSE) that surrounds the ovary, the theca cells, and the granulosa cells (GCs), which essentially reside within the ovarian follicle’s avascular space [2] (Additional file 1). Although the OSE represents a minute fraction of the cell mass of the ovary, evidence shows that OSE is a multipotential epithelium with stem-cell characteristics and plays an important role in tumorigenesis and oogenesis [3,4]. In human-assisted reproduction programs, follicular fluid fills the antrum and surrounds the oocyte. Besides oocytes, the aspirated follicular fluid contains GCs, thecal cells, and ovarian surface epithelial cells. Among follicular cells, GCs show the most common type of cells [5,6], and theca cells were also isolated in follicular fluid [7,8]. Recently, methods have been developed to culture GCs over prolonged time periods and with large quantities of GCs. Kossowska-Tomaszczuk and colleagues first indicated that GCs collected from the follicular fluid had stem cell potential multipotency. They demonstrated that luteinizing GCs isolated from the ovarian follicles of infertile patients included in the assisted reproduction program can be differentiated into three germ cell types, including neurons, chondrocytes, and osteoblasts [9]. However, scant attention has been shown to epithelial cells in follicular fluid.

The present article is the first to describe how epithelial cells could be isolated from human ovarian follicular fluid, and that a subpopulation of these epithelial cells has germline stem cell (GSC) characteristics. Intriguingly, these epithelial cells can form oocyte-like cells spontaneously *in vitro*. We also report that cell colonies from epithelial cells express stem cell multipotency markers and have been successfully differentiated into various somatic cell types from all three germ layers: mesoderm, ectoderm, and endoderm. We also observed that these epithelial cells could be differentiated directly to functional hepatocyte-like cells, which do not exist in ovarian tissues.

## Materials and methods

### Sample collection

Follicular fluid (~5 ml) was obtained from 20 regularly menstruating healthy women undergoing *in vitro* fertilization (IVF), due to tubal factor infertility, from the IVF Center in the International Peace Maternity and Child Health Hospital (Shanghai, China). All women underwent a long IVF protocol (combination of gonadotropin-releasing hormone agonist and recombinant follicle-stimulating hormone) to achieve controlled ovarian hyperstimulation. Follicular fluid was collected from the dominant follicles (>18 mm) during transvaginal ultrasound-guided oocyte aspiration 34 to 36 hours after human chorionic gonadotropin (10,000 IU) administration. Follicular fluid samples used for analysis were macroscopically clear and not contaminated with blood. The samples were centrifuged at 4,000 × *g* at room temperature for 5 minutes to remove supernatants. The pellet-containing cells were resuspended for culturing. Additionally, ovarian tissue biopsies for RT-PCR were obtained from three women with regular menstrual cycles who were undergoing benign gynecological surgery unrelated to an ovarian condition. This work has been approved by the Institutional Ethics Committee of the International Peace Maternity and Child Health Hospital, and written informed consent was obtained from all participants.

### Human follicular epithelial cell culture and colony culture

After the supernatant was discarded, the cellular components were grown in Dulbecco’s modified Eagle’s medium/F12 medium (Gibco, Grand Island, NY, USA) containing 15% fetal bovine serum (Gibco), 1% glutamine, and 1% penicillin/streptomycin (Gibco), supplemented with basic fibroblast growth factor (4 ng/ml; Invitrogen, Carlsbad, CA, USA) at 37°C in a 5% carbon dioxide atmosphere. After being cultured for 5 to 7 days, two kinds of cells grew, including epithelial cells and GCs. The GCs were scraped away to an additional plate for culturing.

After 14 days of culturing, cell colonies developed and were picked up to be maintained on the human amniotic epithelial cell (hAEC) feeder layer [10,11], with ES medium usually used to culture human embryonic stem cells (hESCs) and composed of basal medium: 20% KnockOut Serum Replacement (Gibco), 1 mM l-glutamine (PAA, Cölbe, Germany), 1% nonessential amino acids (PAA, Cölbe, Germany), 0.1 mM 2-mercaptoethanol (Invitrogen), 13 mM HEPES, and 8 ng/ml human basic fibroblast growth factor (Invitrogen).

### *In vitro* differentiation assay

Cell clones were picked and cultured under feeder-free conditions in embryoid body stander medium (Millipore, Billerica MA, USA) to develop into embryoid bodies. The embryoid bodies were then transferred to gelatin-coated plates for further spontaneous differentiation for 5 to 10 days. Cells were then fixed with 4% paraformaldehyde for 30 minutes and permeabilized for an additional 10 minutes with 0.1% Triton X-100 (Sigma, St. Louis,MO,USA). The blocking step was 30 minutes with 2% fetal bovine serum in phosphate-buffered saline (PBS). Cells were incubated with antibody against nestin (mouse anti-human, 1:200; Santa Cruze Biotechnology,Santa Cruze,CA,USA), anti Sox-17 (rabbit anti-human, 1:200; Santa Cruz), and brachyury (mouse anti-human, 1:200; Santa Cruz) for 2 hours. Each antibody was detected using corresponding secondary antibodies conjugated to fluorescein isothiocyanate.

### Hepatocyte differentiation and function analysis

For hepatocyte differentiation, cells were placed at the density of 1.5 × 10^3^ cells/cm^2^ on collagen I-coated plates. At day 1, the medium was changed to stage I differentiation medium: Iscove’s modified Dulbecco’s medium with 20 ng/ml hepatocyte growth factor (Invitrogen), 10 ng/ml fibroblast growth factor 4 (Invitrogen), 1% insulin-transferrin-selenium (ITS premix, Invitrogen) and 5 mM nicotinamide (Invitrogen). At day 9, the medium was changed to stage II differentiation medium (Iscove’s modified Dulbecco’s medium with 20 ng/ml Oncostatin M, 1 μM dexamethasone, 1% ITS premix), and cells were incubated in this medium for 8 days to generate hepatocyte-like cells [12].

For function analysis, cells were stained by Periodic Acid Schiff (Sigma) following the manufacturer’s instructions. For the indocyanine green (Sigma) uptake assay, cell medium was changed to 1 mg/ml indocyanine green and incubated at 37°C for 1 hour, followed by washing with PBS three times.

### Flow cytometry

A total of 1 × 10^6^ epithelial cells were suspended in 2% bovine serum albumin/PBS and labeled with DEAD box polypeptide 4 (DDX4) antibody (polyclonal antibodies against the carboxyl terminus, ab13840; Abcam, Cambridge, UK) and were evaluated using a FC500 flow cytometer (Beckman Coulter, Miami, FL, USA) gated against negative controls (unstained cells and cell fractions processed without primary antibody). Data were analyzed by Beckman Coulter CXP software.

### Dual immunofluorescence-based detection of BrdU and DDX4

To assess proliferation, epithelial cells were treated with 10 μM bromodeoxyuridine (BrdU; Sigma) for 48 hours, and then cells were fixed with 4% paraformaldehyde for 20 minutes at room temperature and permeabilized with 0.1% Triton X-100 for 10 minutes at room temperature. Cells were then blocked with blocking solution for 30 minutes and incubated with anti-BrdU (mouse anti-human 1:200, Lab Vision Corporation) and anti-DDX4 (rabbit anti-human 1:00, ab13840; Abcam, Fremont, CA, USA) for 1 hour at room temperature. Cells were then probed with fluorescein isothiocyanate-labeled IgG (1:200; Santa Cruz). Fluorescence images were obtained with a Leica DMI3000 microscope (Heidelberg, Germany).

### Immunofluorescence staining

Epithelial cells, oocyte-like cells, and colonies maintained on hAECs were fixed with 4% paraformaldehyde for 15 to 20 minutes at room temperature and permeabilized with 0.1% Triton X-100 for 10 minutes at room temperature. Cells were then blocked with blocking solution for 30 minutes and incubated with anti-OCT4 (rabbit anti-human 1:200; Santa Cruz), anti-NANOG (rabbit anti-human, 1:200; Chemicon, Rolling Meadows, IL, USA), anti-DAZL (goat anti human, 1:500; Santa Cruz), anti-STELLA (goat anti-human, 1:200; Santa Cruz), anti-ZPC (rabbit anti-human, 1:200; Santa Cruz), anti-SCP3 (rabbit anti-human, 1:800; Abcam), anti-GDF9 (rabbit anti-human, 1:200; Millipore), anti-SSEA4 (mouse anti-human, 1:100; Millipore), anti Tra-1-60 (mouse anti-human, 1:100; Millipore), and anti Tra-1-81(mouse anti-human, 1:100; Millipore) antibody for 1 hour at room temperature. Cells were then probed with fluorescein isothiocyanate-labeled IgG (1:200; Santa Cruz) or Rodamine (TRITC)-labeled IgG (1:100; Invitrogen) and incubated at room temperature for another 20 minutes. The slides were then covered with mounting medium (glycerol diluted 3:1 in PBS; Vector Laboratories, Burlingame, CA, USA). Fluorescence images were obtained with a Leica DMI3000 microscope.

### RNA extraction and real-time quantitative PCR analysis

Epithelial cells cultured for 1 week, epithelial cells cultured for 1 month, the human skin fibroblast cell line (purchased from Cell bank of China, Shanghai, China) and human ovarian cortex were collected for real-time PCR analysis. Total RNAs were isolated from samples using an RNeasy Mini Kit (Qiagen, Chatsworth, CA, USA) and 500 ng total RNAs were used in reverse transcription with an iScript cDNA synthesis kit (Bio-Rad, Hercules, CA, USA). Real-time quantitative RT-PCR was performed on cDNA using IQ SYBR Green (Bio-Rad) on the Mastercycler® ep realplex (Eppendorf, Hamburg, Germany). All reactions were performed in a 25 μl volume. Primer sequences are presented in Additional file 2. Reaction conditions for NANOG, octamer-binding transcription factor 4 (OCT4), sex determining region Y-box 2 (SOX2), and TERT were: 94°C for 2 minutes, then 94°C for 30 seconds, 60°C for 30 seconds, and 72°C for 45 seconds for 28 cycles, and then 72°C for 10 minutes. Conditions for deleted in azoospermia-like (DAZL), developmental pluripotency-associated protein 3, stella-related protein (STELLA), B-lymphocyte-induced maturation protein 1 (BLIMP1), stimulated by retinoic acid gene 8 (STRA8), probable ATP-dependent RNA helicase DDX4, vasa homolog (VASA), growth and differentiation factor 9 (GDF9), syntaptonemal complex protein SCP3, zona pellucida gene family ZPA and ZPC, and 18s RNA were: 94°C for 2 minutes, then 94°C for 30 seconds, 53°C for 30 seconds, and 72°C for 45 seconds for 28 cycles, and then 72°C for 10 minutes.

### Time-lapse analysis

Images were acquired using an inverted Olympus IX81 Microscope (Olympus, Tokyo, JP) equipped with temperature control components. Time-lapse images were acquired every 3 minutes in two different focal planes over 20 hours of culture using a 40× oil-immersion objective.

### Teratoma formation test

The colonies derived from epithelial cells were examined for their ability to form teratomas *in vivo*. Then 2 × 10^6^ cells were mixed with Matrigel and injected subcutaneously into nonobese diabetic/severe combined immunodeficiency (SCID) mice, which were then monitored weekly for up to 6 months for tumor formation. H9 hESCs were used as positive controls.

### Statistical analysis

Means for relative gene expression were compared by analysis of variance using Microsoft Excel software (FineExcel, v3.3, China). Statistical significance was set at *P* <0.05 or *P* <0.01.

## Results

### Epithelial cells in human follicular fluid

Follicular fluid samples were collected from 20 patients (mean age ± standard deviation, 34.0 ± 5.2 years; range, 26 to 40 years) who were undergoing oocyte pick up for IVF due to tubal factor infertility. We were able to establish epithelial cell cultures from 18 samples, but the other two cases failed due to serious blood contamination.

**Figure 1:**
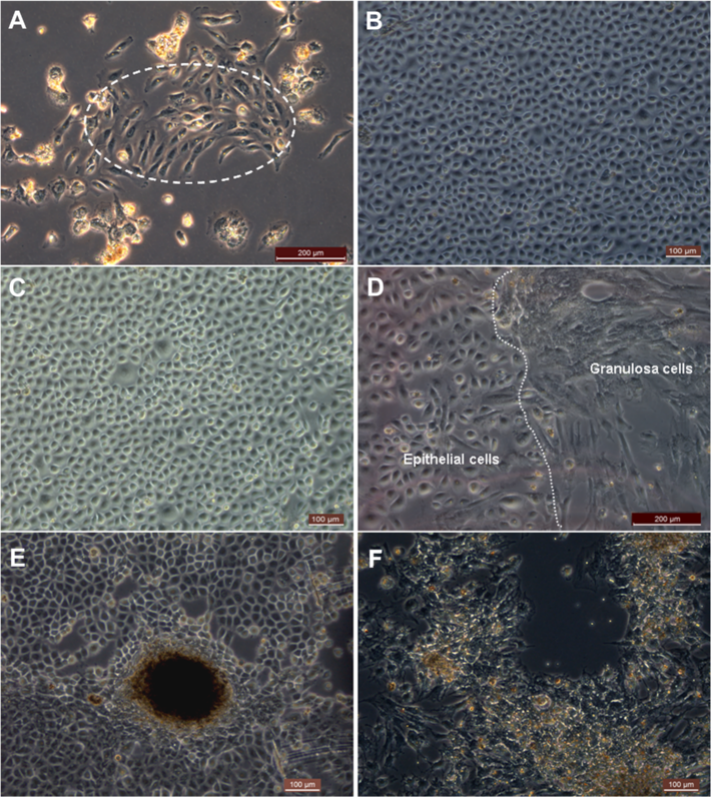
**Morphology of epithelial cells in follicular fluid. (A)** Epithelial cells (inside dotted line) were found from one sample of follicular fluid after 3 days of culturing. **(B)** Epithelial cells had cobblestone-like morphology and grew rapidly after 7 days of culturing. **(C)** Epithelial cells were found from another sample of follicular fluid after 5 days of culturing. **(D)** To the left of the dotted line were epithelial cells and to the right were granulosa cells. **(E)** Human embryonic stem cell-like colonies were growing in the epithelial cell culture. **(F)** Contrasting with epithelial cells, granulosa cells appeared irregularly polygonal, and the pseudopodia were long and tightly adherent. Scale bars: 200 μm **(A, D)** and 100 μm **(B, C, E, F)**.

**Figure 2:**
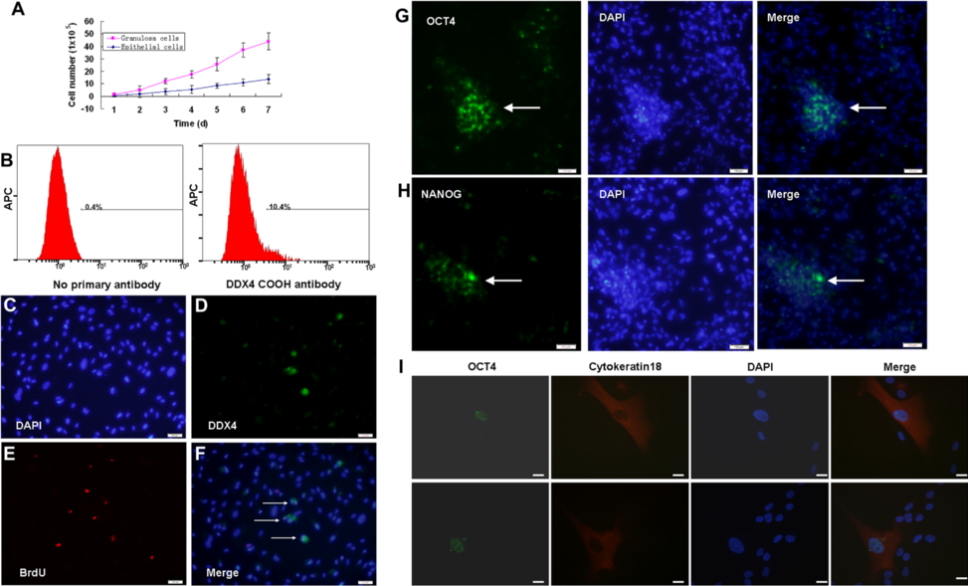
**Stem cell characteristics of follicular fluid-derived epithelial cells. (A)** Typical cell growth curve of epithelial cells after seeding 2.5 × 10^4^ cells in each well of 24-well culture plates, compared with granulosa cells. **(B)** Cell-surface expression of DDX4 in epithelial cells was detected by fluorescence-activated cell sorting analysis after 14 days of propagation. **(C), (D), (E), (F)** Assessment of epithelial cells proliferation by dual detection of DDX4 expression (green) and bromodeoxyuridine (BrdU) incorporation (red) *in vitro* cultures. **(G), (H)** Part of the epithelial cells was OCT4-positive or NANOG-positive. **(I)** Double staining of OCT4 and cytokeratin 18 in epithelial cells. Scale bars: 100 μm **(C, D, E, F, G, H)** and 20 μm **(I)**. DAPI, 4′,6-diamidino-2-phenylindole.

During the first 3 days of culturing, small cuboidal epithelial cells were found and grew only slightly (from 8 to 10 μm; Figure 1A). After 7 days of culturing, they started to grow densely (Figure 1B,C). At the same time, the GCs grew faster than the epithelial cells (Figures 1D,F and 2A). To prevent GC overgrowth, we scraped the GCs off to help the epithelial cells grow. The epithelial cells then grew well, and cell colonies were formed that resembled early embryonic stem cell colonies (Figure 1E).

### *In vitro* characterization of epithelial cells derived from human follicular fluid

The epithelial cells required passage at a confluence of every 5 to 6 days, with an estimated doubling time of 60 hours, whereas the doubling time of GCs was 24 hours (Figure 2A). GSCs, which expressed exclusively DDX4 protein, have successfully been isolated recently from ovaries of neonatal and adult mice as well as from human ovaries [13-15]. We hypothesized that GSCs might also exist in human follicular fluid. To identify and confirm their presence, a rabbit polyclonal antibody against the C terminus of DDX4 protein according to White and colleagues was used for fluorescence-activated cell sorting analysis [13]. Results showed that DDX4 cell-surface expression after fluorescence-activated cell sorting was detectable on 10.4% of the epithelial cells’ surfaces after 7 days of propagation (Figure 2B). Cell proliferation analysis was carried out by adding BrdU to the culture medium. Furthermore, a dual analysis of DDX4 expression and BrdU incorporation in epithelial cell cultures derived from human follicular fluid revealed a few cells that were double positive for DDX4 and BrdU (Figure 2C,D,E,F), which demonstrates that these cells were actively dividing.

To confirm the epithelial cells’ stem cell characteristics, we performed an immunofluorescence analysis of OCT4 and NANOG markers, and the results showed that a few epithelial cells were stained by these two marker genes (Figure 2G,H,I,J,K,L). Further, Figure 2I shows that early epithelial cells co-expressed OCT4 and epithelial marker cytokeratin 18.

### Spontaneous differentiation of epithelial cells into oocyte-like structures

**Figure 3:**
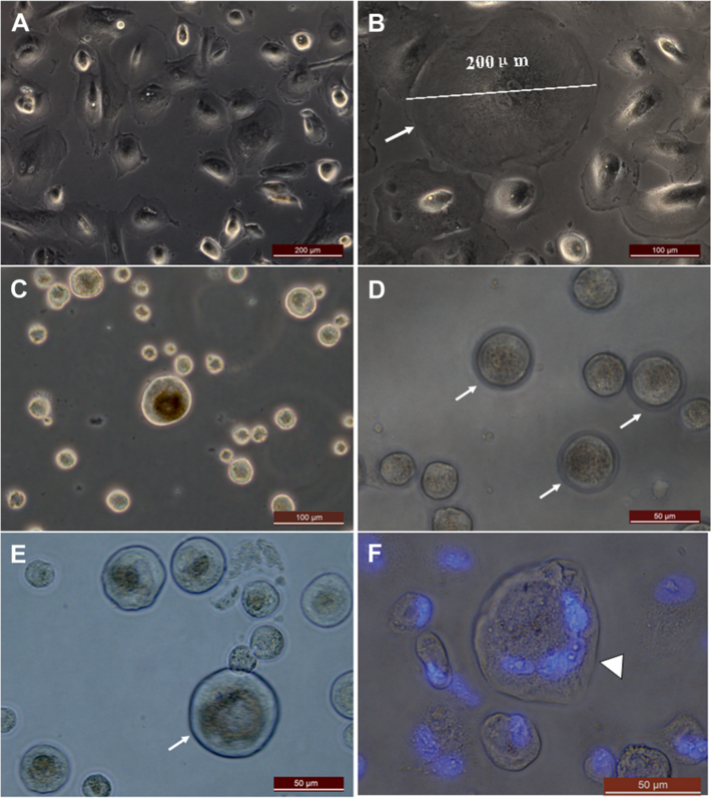
**Larger oocyte-like structures developed spontaneously from epithelial cells derived from human follicular fluid. (A)** The volume of primary epithelial cells increased after 2 weeks of culturing. **(B)** Prominent nucleus and perinuclear accumulation of cytoplasmic organelles were present in large oocyte-like cells. Different sizes of oocyte-like structures after they were detached by trypsin after **(C)** 2 weeks , **(D)** 3 weeks , and **(E)** 4 weeks of culturing**. (F)** Blastocyst-like structures stained with 4′,6-diamidino-2-phenylindole developed after 6 weeks of culturing (arrowhead). Zona pellucida-like structures (arrow) were observed in epithelial cells post culture, indicating different stages of oocyte-like structure maturation. Scale bars: 200 μm **(A)**, 100 μm **(B, C)**, and 50 μm **(D, E, F)**.

During cell culturing, the epithelial cells appeared to increase in size and differentiate spontaneously into large oocyte-like structures attached to the plate bottom (Figure 3A,B). When they were detached by trypsin, a few of these oocyte-like structures appeared to be surrounded by distinct zona pellucida-like structures (Figure 3). Oocyte-like structures with an average diameter of 200 μm, comparable with naturally occurring oocytes, and prominent nucleus and perinuclear organelle accumulation were also observed (Figure 3B). Additionally, a blasto-like structure was also observed after 8 weeks of culturing (Figure 3F).

**Figure 4:**
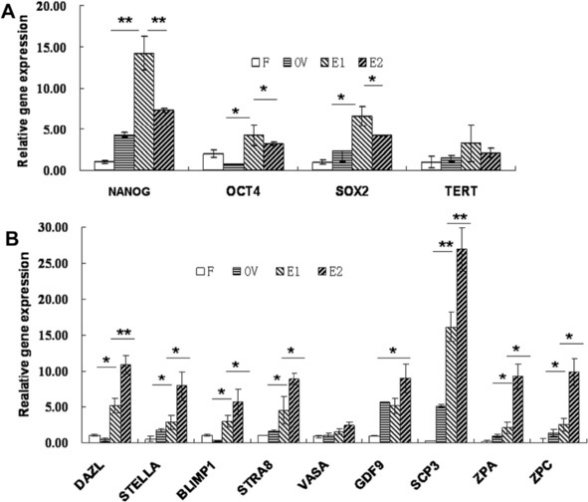
**Real-time PCR analysis of human skin fibroblast, human ovarian cortex, epithelial cells cultured for 1 week, and epithelial cells cultured for 1 month. (A)** Gene expression of transcripts for pluripotency markers showed higher expression levels for OCT4, Nanog, TERT, and Sox-2 in earlier epithelial cells, and decreased after 1 month of culturing. Human ovarian cortex also showed the presence of transcripts for pluripotency markers. **(B)** Transcripts for germ cell markers DAZL, STELLA, BLIMP1, STRA8, VAZA, GDF9, SCP3, ZPA, and ZPC in epithelial cells increased significantly after culturing for 1 month. Comparatively, human ovarian cortex also showed different expression levels of transcripts for germ cell markers. Human skin fibroblast cells served as negative controls, and 18s RNA as a house-keeping control gene was detected in all samples. Data represent mean ± standard error of three independent experiments, *P <0.05, **P <0.01. F, human skin fibroblast; OV, human ovarian cortex; E1, epithelial cells cultured for 1 week; E2, epithelial cells cultured for 1 month.

To understand the possibility that epithelial cells derived from follicular fluid have the potential to differentiate oocyte-like cells, germ cell-specific markers were assayed. First, real-time PCR analyzed epithelial cells with different culturing times. Pluripotent transcripts for NANOG, OCT4, and SOX2 were higher in earlier epithelial cells (1 week of culturing) and decreased after 1 month of culturing (Figure 4A, *P* <0.05). Germ cell markers, including DAZL, STELLA, BLIMP1, STRA8, GDF9, SCP3, ZPA, and ZPC transcripts, increased in postcultured epithelial cells after 1 month of culturing (Figure 4B, *P* <0.05).

**Figure 5:**
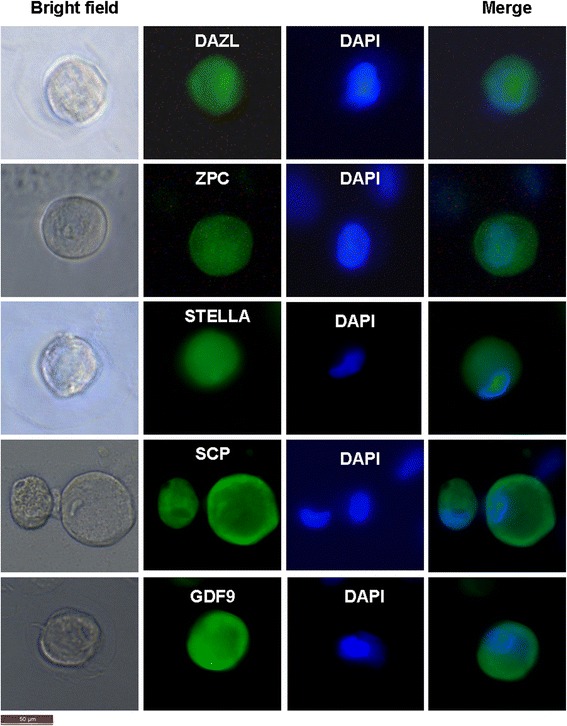
**Characterization of oocyte-like structures observed post culture of epithelial cells by immunolocalization of germ cell markers.** The oocyte-like structures stained positive after 4 weeks culturing for DAZL, ZPC, STELLA, SCP, and GDF-9, respectively. All markers are specific to ooplasm. 4′,6-Diamidino-2-phenylindole (DAPI) was used to counterstain and observe the nuclei. Scale bar = 50 μm.

The oocyte-like structures obtained post culture exhibited positive cytoplasmic staining for DAZL, ZPC, STELLA, SCP, and GDF-9 (Figure 5). The adjacent somatic fibroblasts were negative, indicating specific localization in the germ cells. Respective negative controls with omission of primary antibody were also employed, which showed absent staining (data not shown).

### Time-lapse imaging demonstrates the self-renewal of epithelial cells

**Figure 6:**
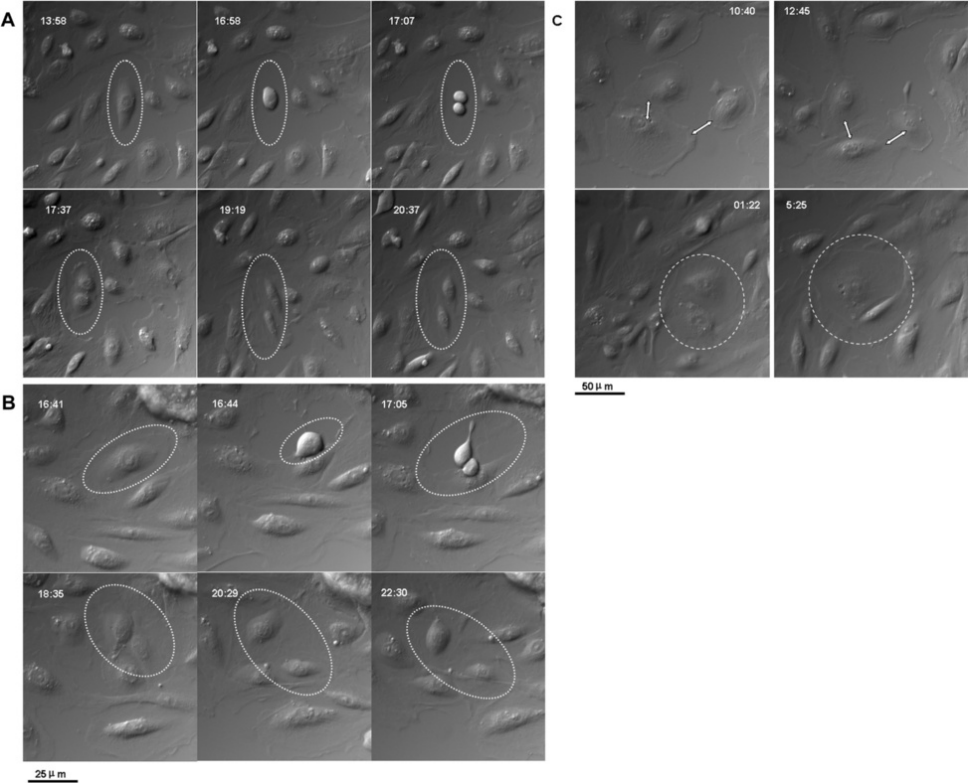
**Self-renewal of epithelial cells and** ***in vitro*** **development of oocytes. (A)** Epithelial cells were split symmetrically. **(B)** Epithelial cells were split asymmetrically. **(C)** Cross-talk between epithelial cells with other cells. Scale bar: 25 μm **(A, B)** and 50 μm **(C)**.

To investigate cell division patterns and the movement and shape of epithelial cells, we analyzed images of epithelial cells originated from human follicular fluid using a computer-based time-lapse acquisition system attached to a differential interference contrast microscope. The epithelial cells showed a tendency to proliferate and migrate and retained contact with neighboring cells during cell division. The cells became rounded, moved up, and started to shake and ascend to disseminate to the substrate to complete the cell division split, including symmetric and asymmetric division. The epithelial cells were split into two cells symmetrically in 6 hours and 39 seconds and were split into two cells asymmetrically in 5 hours and 49 seconds. The epithelial cells had a round-up time of over 6 hours (Figure 6A,B).

Additionally, some cells did not undergo cell division, but these cells contacted neighboring cells through amoeba movement. Their volume increased gradually and oocyte-like structures formed spontaneously (Figure 6C).

### Epithelial cells form embryo stem-like cell colonies which have the pluripotency of stem cells, and could be differentiated directly to functional hepatocyte-like cells

Cell colonies were found in 56% (10/18 samples) of epithelial cell cultures, which resembled early hESC colonies (Figure 1H). When using hAECs [10,11] as a feeder layer, these colonies could be maintained and proliferated with undifferentiated growth. We were able to maintain cell colonies from one sample in undifferentiated conditions for more than 20 passages.

**Figure 7:**
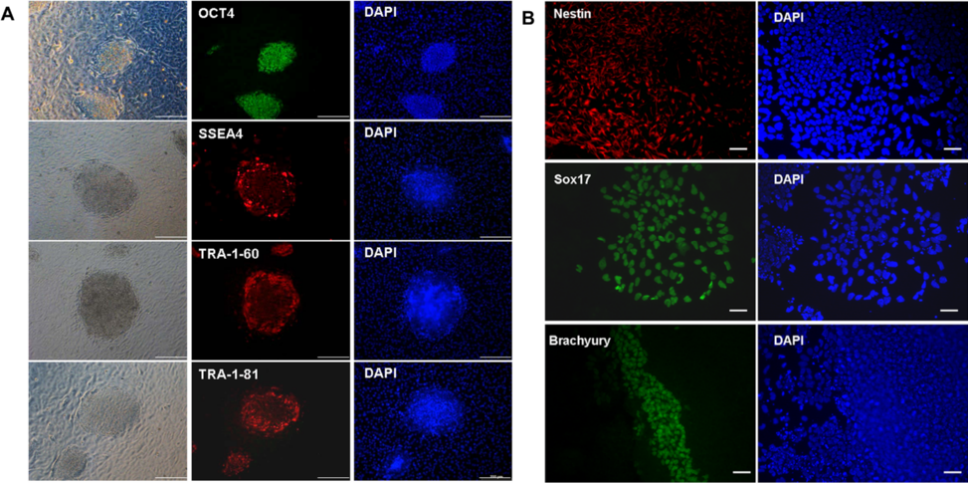
**Pluripotent characteristics of cell colonies derived from epithelial cell cultures using human amnion epithelial cells as a feeder layer. (A)** Cell colonies showed positive immunofluorescence staining for pluripotency markers, namely OCT-4, SSEA4, Tra-1-60, and Tra-1-82. All cell colonies were from the same 2-month-old cell culture. **(B)** Cells were pluripotent, as demonstrated by their potential to differentiate *in vitro* into progeny representing the three germ lineages. Immunofluorescence staining showed differentiated cells expressing nestin (ectoderm), brachyury (mesoderm), and sox17 (endoderm). Scale bars: 200 μm **(A)** and 100 μm **(B)**. DAPI, 4′,6-diamidino-2-phenylindole.

The formed cell colonies were tested for various pluripotency marker expressions: OCT4, stage-specific embryonic antigen-4 (SSEA4), TRA-1-60, and TRA-1-81. Positive staining was observed for cell colonies maintained on hAECs at the 5th passage (Figure 7A).

We then assayed the potential of these cell colonies to differentiate into the three germ layers. Cell colonies were picked and allowed to differentiate in suspension in differentiating medium and form embryoid bodies. The embryoid bodies were then transferred to gelatin-treated plates and grown for another 8 days. Figure 7B shows that these cells were specifically stained with antibodies against sox17 (endoderm), brachyury (mesoderm), and nestin (ectoderm), indicating that differentiated cells from these colonies expressed representative markers of three germ layers.

**Figure 8:**
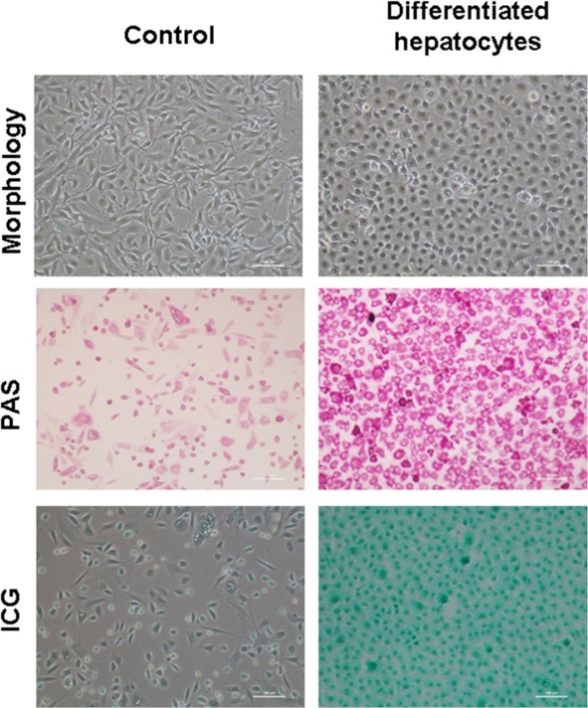
**Differentiation of epithelial cells to functional hepatocyte-like cells.** Glycogen storage by hepatocyte-like cells was confirmed by Periodic Acid Schiff (PAS) staining and indocyanine green (ICG) uptake in hepatocyte-like cells (green). Scale bar, 100 μm.

To further analyze the functional stem cells derived from epithelial cells, we directly differentiated our cells to hepatocyte-like cells, and the differentiated cells showed the typical hepatic morphology. Intriguingly, those hepatocyte-like cells displayed typical functions of mature hepatocytes, such as glycogen storage and indocyanine green absorption (Figure 8).

Karyotype analysis demonstrated that chromosomal stability could be maintained after sequential propagation for 20 passages. Normal, stable karyotype is shown in Additional file 3. Additionally, there was no solid tumor formation after injecting cell colonies derived from epithelial cells into SCID mice. However, in all injected control mice, teratoma developed after injecting H9 hESCs (Additional file 4).

## Discussion

Follicular fluid accumulation into the follicle antrum is important for follicular development and oocyte maturation. Evidence suggests that follicular fluid is not only rich in proteins (such as steroids, growth factors, and other peptidergic factors) but also cells [16-18]. Among follicular cells, granulosa and thecal cells were well identified and characterized [7-9]. Epithelial cells theoretically exist in follicular fluid, because the OSE surrounds follicles as oocytes grow and follicles expand (Additional file 1). To our knowledge, this is the first report on the development of epithelial cells in follicular fluid retrieved in the IVF program. This is important for clinical practice because it is a potential stem cell source and an excellent folliculogenesis model.

The OSE is an important human ovary structure. OSE cells differentiate from peritoneal mesothelial cells through their transformation from mesenchymal into epithelial cells and are involved in both reproductive functions and ovarian tumor formation [19]. OSE is reportedly the source of neo-oogenesis [20]. Bukovsky and colleagues observed that OSE cells could be the bipotent source for germ cells and GCs [21]. Furthermore, Virant-Klun and colleagues demonstrated evidence of putative stem cell presence in the OSE layer of postmenopausal women and women with premature ovarian failure [22]. A small population of SSEA4-positive putative stem cells with germline characteristics was also isolated from the OSE of postmenopausal human ovaries devoid of oocytes and is able to produce oocyte-like structures *in vitro* [23-25].

In this study, we isolated from the follicular fluid a population of cuboidal epithelial cells with a diameter from 8 to 10 μm, which have not been defined until now. The growth pattern is different from that of GCs (Figures 1 and 2A). Recently, GSCs have successfully been isolated from ovaries of neonatal and adult mice as well as from human ovaries, which challenges the viewpoint that the bank of ovarian oocytes is not renewed in postnatal female mammals [14,15,26]. Herein, we also found that a small population of these epithelial cells expressed marker characteristic for germline stem cells, such as OCT4, NANOG, and DDX4 (Figure 2). Our results are in accordance with White and colleagues in that the rare cells with cell-surface DDX4 expression present in the ovaries of reproductive-age women represent adult human ovarian stem cells [13].

In the epithelial cell culture system, oocyte-like cells developed spontaneously. They reached diameters up to 75 to 200 μm, which is comparable with human oocytes in the IVF program. Some of the oocyte-like cells developed a zona pellucida-like structure and expressed DAZL, STELLA, ZPC, SCP, and GDF9 transcription markers. Some oocyte-like cells grew and even reached a diameter of 120 μm. Blasto-like structures could be found in cultured epithelial cells. Unfortunately, most of the oocyte-like cells were aging. Rarely observed were germinal vesicle-like structures and extruded polar body-like structures. However, Virant-Klun and colleagues indicated that germinal vesicle oocyte-like cells could be found in OSE culture from postmenopausal women [23]. The *in vitro* oogenesis culture condition is very complicated and needs to be further optimized.

Using time-lapse photography in epithelial cell culture, we observed that *in vitro* developing oocyte-like structures are acquired by accompanying fibroblast-like cells or exploit adjacent satellite cells (Figure 6C). Images from time-lapse photography show that early developing oocyte-like structures have low optic density. We also first reported epithelial cell self-renewal. Both symmetric and asymmetric division split of cells was observed (Figure 6A,B). Bukovsky and Caudle reported that asymmetric divisions were observed in ovarian stem cells in human fetal ovaries accompanied by a diminution of MHC-I and light chain expression in one of the daughter cells. The size of these cells substantially increases compared with typical ovarian stem cells. Such cells resemble intraepithelial germ cells and subsequently divide symmetrically [27,28]. So far, we do not have evidence that these divisions are associated with stem cells.

Evidence also showed that human OSE stem cells retain embryonic stem cell characteristics. Virant-Klun and colleagues isolated a population of small round cells with a bubble-like structure and a diameter from 2 to 4 μm from OSE scrapings in 21 postmenopausal women whose ovaries contained no follicles. Fascinatingly, these small cells were transformed into oocyte-like cells and exhibited positive staining for some pluripotent embryonic stem cell markers such as SSEA-4, Oct-4, Sox-2, and NANOG [23]. These findings were further confirmed by Parte and colleagues after scraping adult human OSE. These stem cells underwent spontaneous differentiation into oocyte-like structures, parthenote-like structures, embryoid body-like structures, cells with neuronal-like phenotypes, and embryonic stem cell-like colonies. The epithelial cells transformed into mesenchymal phenotypes by epithelial–mesenchymal transition were also observed in OSE culture [29]. Recently, Stimpfel and colleagues demonstrated that stem cells originating from adult human ovarian cortex formed colonies and expressed pluripotency/multipotency markers. These colonies were able to differentiate *in vitro* into various somatic cell types in all three germ layers. However, these cells did not form teratoma when injected into immunodeficient mice [30]. Consistent with those previous reports, these epithelial cells formed embryonic-like stem cell colonies and could be maintained and proliferated with undifferentiated growth using hAECs as a feeder layer. These colonies expressed OCT4, SSEA4, TRA-1-60, and TRA-1-81 of pluripotent transcription markers. They had the potential to differentiate into three germ layer cells *in vitro*, but showed no teratoma formation *in vivo*. Intriguingly, these epithelial cells also could be directly differentiated to functional hepatocyte-like cells, which do not exist in ovarian tissues.

We conclude that these epithelial cells derived from follicular fluid are an integral part of the OSE. A small population displayed oocyte cell morphology and pluripotent stem cell expression patterns. This observation provides more evidence for the possibility of *de novo* folliculogenesis and oogenesis in the adult human ovary, which is contrary to the persisting dogma about the end number of follicles and oocytes at birth [31]. In the assisted reproduction program, follicular fluid is unavoidably removed from the antrum during transvaginal ultrasound-guided oocyte aspiration from mature follicles. Following oocyte removal, the remaining cell-rich follicular aspirate is usually discarded in daily practice. However, it could be used as a potential stem cell source. The epithelial cells derived from ovarian follicular fluid may provide a promising technical tool for *in vitro* maturation of both human and animal ovarian follicles and can be used in reproductive toxicology, drug targeting, assisted reproduction, and regenerative medicine in the future.

## Conclusion

In summary, the epithelial cell culture could be successfully established from human follicular fluids. A small population of epithelial cells has stem cell characteristics and is a potential stem cell source. The epithelial cells derived from ovarian follicular fluids may provide a promising technical tool in oogenesis and regenerative medicine in the future.

## Additional files

Additional file 1:
**Figure S1.** Showing the OSE surrounding the antral follicle as oocytes grow and follicles expand. Additional file 2:
**Table S1**. Presenting the PCR primer sequences. Additional file 3:
**Figure S2.** Showing that karyotype analysis showed a normal chromosomal complement (46, XX) in epithelial cell colonies derived from follicular fluid after passage 20. Additional file 4:
**Figure S3.** Showing no teratoma formed after injecting epithelial cell colonies into SCID mice, whereas teratoma formed after injecting hESC line H9 into SCID mice. The teratoma contained tissue components of all three embryonic germ cell layers, including the endoderm (A, intestinal mucosa), mesoderm (B, cartilage), and ectoderm (C, neuron epithelium). Original magnification × 100.

## Abbreviations

BLIMP1: B-lymphocyte-induced maturation protein
BrdU: bromodeoxyuridine
DAZL: deleted in azoospermia-like
DDX4: DEAD box polypeptide 4 (also commonly VASA homologue)
GC: granulosa cell
GDF9: growth and differentiation factor 9
GSC: germline stem cell
hAEC: human amniotic epithelial cell
hESC: human embryonic stem cell
IVF: *in vitro* fertilization
OCT4: octamer-binding transcription factor 4
OSE: ovarian surface epithelium
PBS: phosphate-buffered saline
SCID: severe combined immunodeficiency
SCP: syntaptonemal complex protein
SOX2: sex determining region Y-box 2
SSEA4: stage-specific embryonic antigen-4
STELLA: developmental pluripotency-associated protein 3, stella-related protein
STRA8: stimulated by retinoic acid gene 8
VASA: probable ATP-dependent RNA helicase DDX4, vasa homolog
ZPA: zona pellucida gene family A
ZPC: zona pellucida gene family C
