# Supplementary figures and images for: Identification and characterization of epithelial cells derived from human ovarian follicular fluid
Source: Stem Cell Res Ther. 2015 Feb 20;6(1):13. doi: 10.1186/s13287-015-0004-6 (PMC4392788; doi:10.1186/s13287-015-0004-6)

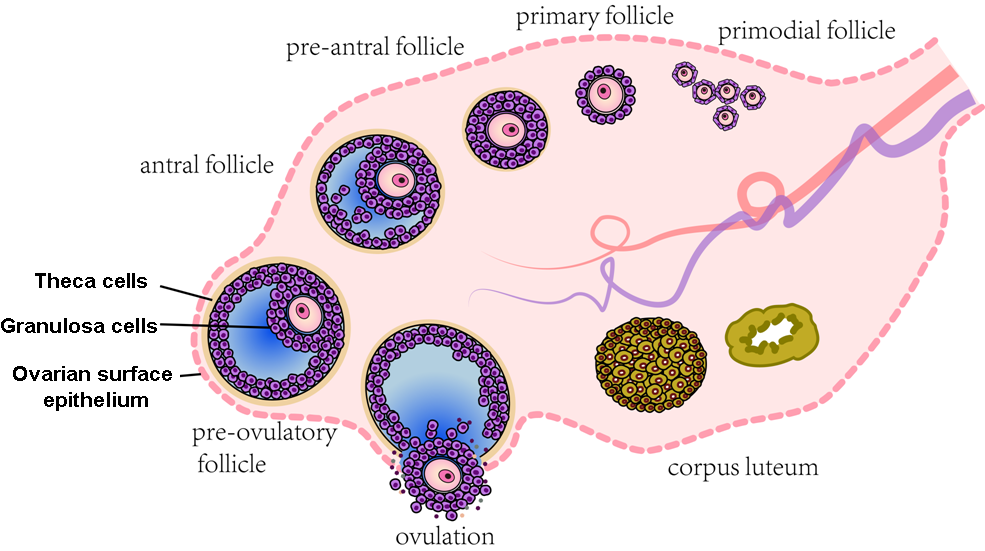

Supplement: Additional file 1: — Figure S1. Showing the OSE surrounding the antral follicle as oocytes grow and follicles expand. [file 13287_2015_4_MOESM1_ESM.tiff]

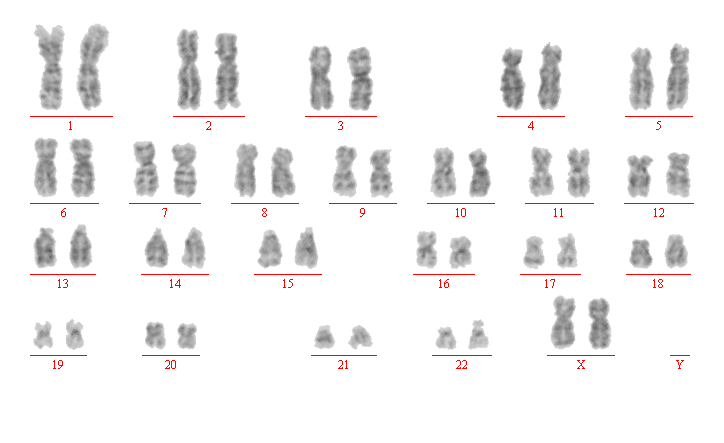

Supplement: Additional file 3: — Figure S2. Showing that karyotype analysis showed a normal chromosomal complement (46, XX) in epithelial cell colonies derived from follicular fluid after passage 20. [file 13287_2015_4_MOESM3_ESM.tiff]

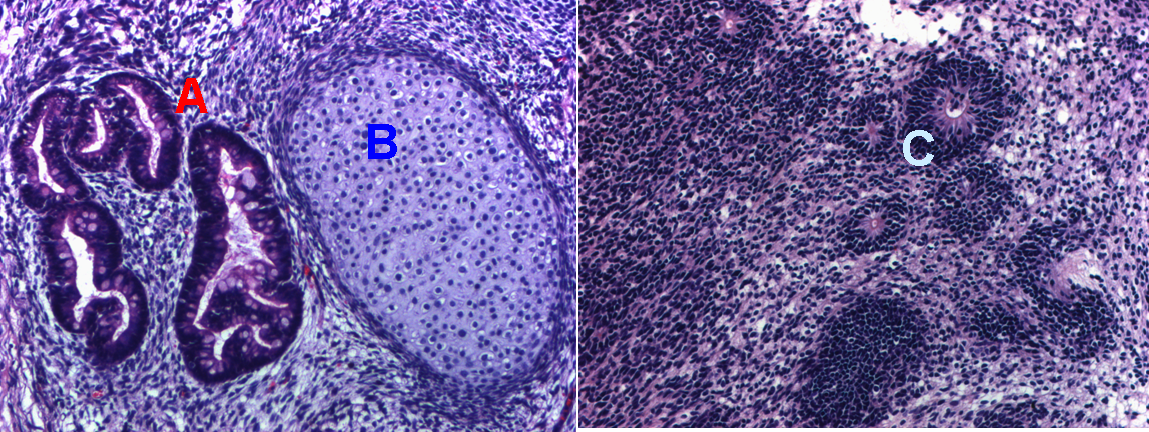

Supplement: Additional file 4: — Figure S3. Showing no teratoma formed after injecting epithelial cell colonies into SCID mice, whereas teratoma formed after injecting hESC line H9 into SCID mice. The teratoma contained tissue components of all three embryonic germ cell layers, including the endoderm (A, intestinal mucosa), mesoderm (B, cartilage), and ectoderm (C, neuron epithelium). Original magnification × 100. [file 13287_2015_4_MOESM4_ESM.tiff]
