# Supplementary material for: Identification and characterization of epithelial cells derived from human ovarian follicular fluid
Source: Stem Cell Res Ther. 2015 Feb 20;6(1):13. doi: 10.1186/s13287-015-0004-6 (PMC4392788; doi:10.1186/s13287-015-0004-6)
Supplement: Additional file 2: — Table S1. Presenting the PCR primer sequences. [file 13287_2015_4_MOESM2_ESM.doc]

**Supplementary Information**

Table 1. PCR primer sequences

| Gene product | Forward(F) and reverse(R) primers(5’ → 3’) | Size(bp) |
| --- | --- | --- |
| *NANOG* | F: GGGCCTGAAGAAAACTATCCATCC | 400 |
|  | R: TGCTATTCTTCGGCCAGTTGTTTT |  |
| *OCT4* | F: GGCCCGAAAGAGAAAGCGAACC | 224 |
|  | R: ACCCAGCAGCCTCAAAATCCTCTC |  |
| *SOX2* | F: GCGCGGGCGTGAACCAG | 396 |
|  | R: CGGCGCCGGGGAGATACA |  |
| *TERT* | F: AGAGTGTCTGGAGCAAGTTGC | 185 |
|  | R: CGTAGTCCATGTTCACAATCG |  |
| *BLIMP1* | F: AAGTGTAACTCCAGCACTGTG | 290 |
|  | R: CCAAAACGTGTGCCCTTTGGTATG |  |
| *STELLA* | F: CTCAAATCTCCTCCGAGACG | 137 |
|  | R: TTCGATTTCCCTGAGGACTG |  |
| *DAZL* | F: AATGACGTGGATGTGCAGAA | 152 |
|  | R: AACTGTGGTGGAGGAGGATG |  |
| *VASA* | F: TTGGGAAGCAGAAATCAACC | 240 |
|  | R: AAACCACCCATTGTGGATGT |  |
| *STRA8* | F:TCGTCTCCGCGGCCATCTCC | 153 |
|  | R: TGTCCTTCACGCTGCCCTCG |  |
| *ZPA* | F:GCAGCACTTACCTTGCTTCC | 167 |
|  | R: TCTGGTGGTCACAGCTTCAG |  |
| *ZPC* | F:GCAGGCATGTGACAGAAGAA | 106 |
|  | R:GAGGTGTCAGAAGGCAAAGC |  |
| *SCP1* | F: CAAAAGCCCTTTGCATTGTT | 225 |
|  | R: CTCAAACACGGGCAAGAAAT |  |
| *SCP3* | F: TATGGTGTCCTCCGGAAAAA | 238 |
|  | R: AACTCCAACTCCTTCCAGCA |  |
| *GDF9* | F: GCACAGGTACAACCCTCGAT | 122 |
|  | R: GCACTGAGGAGTCCAGCTTC |  |
| *18s RNA* | F: CGTTGATTAAGTCCCTGCCCTT | 202 |
|  | R: TCAAGTTCGACCGTCTTCTCAG |  |
